# Anti–PD-1 Monoclonal Antibody Combined With Anti-VEGF Agent Is Safe and Effective in Patients With Recurrent/Metastatic Head and Neck Squamous Cancer as Second-Line or Beyond Treatment

**DOI:** 10.3389/fonc.2022.781348

**Published:** 2022-02-24

**Authors:** Yonghong Hua, Ruizeng Dong, Ting Jin, Qifeng Jin, Xiaozhong Chen

**Affiliations:** ^1^ Key Laboratory of Radiation Oncology, Zhejiang Cancer Hospital, University of Chinese Academy of Sciences, Hangzhou, China; ^2^ Department of Abdominal Medical Oncology, Zhejiang Cancer Hospital, University of Chinese Academy of Sciences, Hangzhou, China

**Keywords:** head and neck squamous cell carcinoma, anti–PD-1 monoclonal antibodies, anti-VEGF agents, safety, efficacy

## Abstract

**Background:**

Numerous preclinical studies have revealed the complex regulatory mechanisms between anti-angiogenesis and immune inhibition in the tumor immune microenvironment and have proposed the efficacy of combined immunotherapy and anti-angiogenic treatment. Moreover, the combination strategy had been confirmed in a number of clinical trials. In this study, we aimed to evaluate the safety and efficacy of this combination strategy in recurrent/metastatic head and neck squamous cell carcinoma.

**Methods:**

In this real-world study, 43 patients who received the combination of programmed cell death protein 1 (PD-1) inhibitors and anti-vascular endothelial growth factor (VEGF) agents in Zhejiang cancer hospitals between March 2019 and December 2020 were reviewed. Clinical characteristics and follow-up data were collected, and the preliminary efficacy and safety of the combination therapy were assessed.

**Results:**

The median follow-up time was 12.4 months (range, 3.7-25.3 months), and the follow-up rate was 100%. The median duration of exposure was 9.5 months. Thirty-seven patients (86.0%) reported treatment-related adverse events (TRAEs) of any grade. The most frequently reported events were fatigue, decreased appetite, and hypertension. Grade 3 TRAEs occurred in 8 patients (18.6%), and no grade 4 or 5 TRAEs occurred. Twenty-four patients (55.9%) had an overall response to treatment: 6 (14.0%) had a complete response and 18 (41.9%) had a partial response. In addition, 5 (11.6%) patients had stable disease, and the disease control rate 12 was 67.4%. The median time to response was 1.6 months (range, 1.1-2.8 months). The median progression-free survival (PFS) was not reached, and the 1-year PFS rate was 69.1%. The 1-year overall survival (OS) rate was 87.7%. Patients with primary tumors located in the nasopharynx had better OS than those with tumors outside the nasopharynx. ECOG PS were related to PFS; patients with an ECOG PS of 0 had a slight survival advantage.

**Conclusion:**

The combination strategy of anti–PD-1 monoclonal antibodies and anti-VEGF agents was tolerable in patients with recurrent/metastatic head and neck cancer. This treatment exhibited antitumor potential despite the heavily pretreated population.

## Introduction

Head and neck squamous cell carcinoma (HNSCC) include a variety of malignancies in the oral cavity, nasal cavity, oropharynx, nasopharynx, and laryngopharynx. In 2020, more than 870,000 new cases of HNSCC were diagnosed globally, accounting for 4.5% of all newly diagnosed malignant cancers worldwide (1). More than 50% of diagnosed HNSCC cases are at locally advanced stages. Despite the use of multimodal treatment for locally advanced HNSCC, more than 50% of these patients will experience recurrence or metastasis within 3 years (2). EXTREME regimen (cetuximab + cisplatin/carboplatin + fluorouracil) is often used to treat recurrent and/or metastatic (R/M) disease, which has been approved as the first-line choice for R/M HNSCC in many countries (3). However there is no standard treatment for R/M HNSCC that fails to respond to first-line platinum-containing systemic chemotherapy. Single-drug chemotherapy or cetuximab is usually recommended, but the median overall survival (OS) is 7 or fewer months (4).

Immunotherapy has revolutionized the treatment dilemma, enabling durable control of some previously incurable R/M HNSCC (5). Programmed cell death protein 1 (PD-1) is a transmembrane immune checkpoint receptor that is expressed on activated T cells, B cells, natural killer cells, and some myeloid cells; it can recognize PD-1 ligands (PD-L1 and PD-L2) and limit the cytotoxic T cell effect within tissues (6, 7). Overexpression of PD-L1 has been found to block the antitumor immune response in some tumor cells (8). Zandberg et al. reported that PD-L1 was expressed in 50%-60% of HNSCC (9). Anti–PD-1 antibodies can improve survival in some patients with R/M HNSCC by blocking the recognition of PD-1 and PD-L1. The pivotal phase III Checkmate 141 trial and Keynote 040 trial confirmed respectively the efficacy of nivolumab and pembrolizumab in pretreated R/M HNSCC compared with the investigator’s choice of treatment (10, 11). The same benefit was observed in R/M nasopharyngeal carcinoma in the POLARIS-02 study of Toripalimab (12).

On the basis of above clinical researches, PD-1 inhibitors as monotherapy have been approved by the US Food and Drug Administration and the European Medicines Agency to treat some pretreated R/M HNSCC. However, two key problems remain: First, the overall response rate (ORR) remains relatively low—the ORR ranges from 14% to 43% in pretreated R/M HNSCC (including nasopharyngeal cancer)—so the majority of patients with R/M HNSCC do not benefit from this monotherapy (10–12). Second, the therapeutic effect differs significantly across patients. A durable response could last a long time in some patients, whereas some patients could experience hyperprogression (13). No robust mechanistic data has explained the unpredictable clinical response to anti–PD-1 antibodies. Given the problems with immunotherapy as monotherapy, many clinical studies have explored the combination of different immune checkpoint inhibitors or immune checkpoint inhibitors with chemotherapy, radiotherapy, and targeted therapy (14–16).

Anti-angiogenic agents have opened a new window for immunotherapy combinations. Initially, anti-angiogenic agents appeared to play an antitumor role by blocking neovascularization (17). Subsequently, it was found that anti-angiogenic agents could also regulate immune cells and the tumor immune microenvironment (18). Some exploratory studies have verified the value of anti-angiogenic agents combined with immunotherapy in lung cancer, liver cancer, and renal cell cancer (19–22). Keynote-146 (NCT02501096) is an open-label, one-arm phase Ib/II clinical study to evaluate the efficacy of Pembrolizumab combined with Lenvatinib in a variety of solid tumors. 22 patients with R/M HNSCC were included in the cohort. The data showed that the ORR of the combined regimen was 46%, and the median duration of remission was 8.2 months (95% CI:2.2-12.6). The median PFS was 4.7 months (95% CI:4.0-9.8). Based on the good results, we tried to use the both drugs to posterior line treat R/M HNSCC in clinical practices. Most of patients had received multiple systemic chemotherapy. In this real-world study, we collected and analyzed the data about these patients’ characteristics, treatment experience, toxicity and tumor control, and the safety and preliminary efficacy were assessed.

## Methods

### Patient Selection

This real-world study enrolled patients who were diagnosed as R/M HNSCC (including nasopharyngeal cancer) and treated with the combination of PD-1 inhibitors and anti-VEGF agents in Zhejiang cancer hospitals between March 2019 and December 2020. PD-1 inhibitors included camrelizumab (Jiangsu Hengrui Medicine, Lianyungang, China), toripalimab (Junshi Bioscience, Shanghai, China), tislelizumab (Beigene, Guangzhou, China), sintilimab (Innovent Biologics, Suzhou, China), nivolumab (Bristol-Myers Squibb, Newyork, USA), and pembrolizumab (Merck & Co., Kenilworth, USA). Anti-VEGF agents included anlotinib (Chia Tai Tianqing Pharmaceutical, Lianyungang, China), apatinib (Jiangsu Hengrui Medicine, Lianyungang, China), and bevacizumab (Roche Group, Basel, Switzerland). The study was approved by the Ethics Committee of Zhejiang Cancer Hospital, and the informed consent for this retrospective analysis was waived.

### Data Collection

Treatment related adverse events (TRAEs) were one of observation factors, which were documented according to patient chief complaints and abnormal laboratory measures, including blood chemistry, hematology, coagulation, and urinalysis, at baseline and during the treatment period. National Cancer Institute Common Terminology Criteria for Adverse Events, version 4.0 was used to evaluate the severity of TRAEs. Efficacy was another observation factor. ORR, which was defined as a complete or partial response, was assessed during treatment and for 3 months after treatment discontinuation. Disease control rate at 12 weeks (DCR12), which was defined as complete or partial response or stable disease for more than 12 weeks, and duration of response (DOR), which was defined as the time from the first documentation of objective tumor response until the first documentation of objective tumor progression or death from any cause, whichever occurred first. Magnetic resonance imaging scan of the head and neck as well as chest, abdominal, and pelvic computed tomography scans were performed to assess the response to treatment. Measurable and nonmeasurable diseases were evaluated according to the modified RECIST 1.1 for immune-based therapeutics (iRECIST). Observation factors about efficacy also included OS and progression-free survival (PFS). Patients who discontinued treatment because of toxicity without evidence of disease progression had their PFS censored at the time of cutoff.

### Statistical Analysis

Statistical comparisons were performed using chi-squared or Fisher’s exact tests for categorical data and t tests for continuous variables. ORR and DCR12 were estimated using the exact binomial method. OS and PFS were estimated and presented graphically using the Kaplan-Meier method. DOR12 was estimated using the Kaplan-Meier method. Patients were censored at the time of death or last follow-up. All statistical analyses were performed with a 5% alpha risk or 95% confidence interval using SPSS software (version 20.0, Chicago, IL, USA).

## Results

### Patient Population

From March 2019 to December 2020, 43 patients with R/M HNSCC received the combination regimens. At the data cutoff on March 31, 2021, the median follow-up time was 12.4 months (range, 3.7-25.3 months), and the follow-up rate was 100%. All patients received anti–PD-1 immune checkpoint inhibitors, namely camrelizumab (n=17), toripalimab (n=13), tislelizumab (n=8), sintilimab (n=2), nivolumab (n=1), and pembrolizumab (n=2). Patients received the following anti-VEGF agents: anlotinib (n=25), apatinib (n=17), and bevacizumab (n=1). All 43 patients were included in the safety analysis and evaluated for response. Most patients (90.7%) were men, and the median age was 55.0 years (range, 30-80 years). All patients had an ECOG PS of 0-1. Most patients (86.0%) had metastatic disease, of which 31 (72.0%) had metastatic disease alone and 4 (14.0%) had metastatic and recurrent disease. Most patients (76.7%) had received 2or more prior lines of systemic therapy for R/M disease, and 30.2% had received 3or more previous lines of systemic therapy. The reason for changing the treatment included disease progression within 6 months (n=21; 48.8%), progression after 6 months (n=12; 27.9%), and intolerance to the previous line of treatment (n=10; 23.3%). Overall, 46.5% of patients had previously received 1or 2 targeted therapies, including anti-EGFR monoclonal antibodies (n=15) and endostatin (n=5). No patient had received PD-1 inhibitors in previous treatment. Two patients (4.7%) received local radiotherapy to the R/M disease. All patient characteristics are shown in **Table 1**.

**Table 1:** Baseline characteristics of study patients.

Characteristic, n (%)	All patients (n = 43)
Median age (range, year)	55.0 (30-80)
Age (year), n (%)	
≤60	28 (65.1)
>60	15 (34.9)
Sex, n(%)	
Male	39 (90.7)
Female	4 (9.3)
ECOG PS, n (%)	
0	22 (51.2)
1	21 (48.8)
No. of prior systemic regimens, n (%)	
1	10 (23.3)
2	20 (46.5)
≥3	13 (30.2)
Reasons for change the previous-line, n (%)	
Progression within 6 months	21 (48.8)
Progression after 6 months	12 (27.9)
Intolerance to pervious line	10 (23.3)
Primary tumor, n (%)	
Nasopharynx	29 (67.4)
Larynx	5 (11.6)
Hypopharynx	6 (14.0)
Oral cavity	2 (4.7)
Oropharynx	1 (2.3)
Previous target therapy, n (%)	
No	23 (53.5)
Yes	20 (46.5)
Radiotherapy to R/M disease, n (%)	
No	41 (95.3)
Yes	2 (4.7)
R/M at baseline, n (%)	
Recurrence	6 (14.0)
Metastasis	31 (72.0)
Recurrence and metastasis	6 (14.0)
PD-L1 CPS status, n (%)	
≥20	6 (14.0)
1-19	5 (11.6)
<1	3 (7.0)
Unknown	29 (67.4)
EGFR status, n (%)	
Positive	37 (86.1%)
Negative	1 (2.3%)
Unknown	5 (11.6%)

ECOG PS, Eastern Cooperative Oncology Group performance status; R/M, recurrence/metastasis; PD-L1, Programmed cell death-Ligand 1; CPS, Combined positive score; EGFR, epidermal growth factor receptor.

### Treatment Delivery and Compliance

A total of 43 patients enrolled in the real-world study. As of March 31, 2021, 21 patients (48.8%) were off treatment; of these, 17 (80.9%) discontinued both study drugs. The reasons for discontinuing both agents were disease progression (n=9), adverse events (n=1), and patient refusal(n=7). One patient experienced bleeding from the larynx, which was relieved through symptomatic treatment; then, this patient discontinued entire treatment. One patient with complete response terminated both agents when the entire treatment lasted for 2 years. Six patients interrupted therapy because of economic reasons, of which, 5 patients received fewer than 3 cycles of combination regimens. Four patients (25.0%) discontinued anti–PD-1 antibodies (n=3; 18.8%) or anti-VEGF agents (n=1; 6.2%). Two patients experienced adverse events (reactive capillary hyperplasia in 1 patient and increased blood creatinine concentration in 1 patient) before discontinuing the PD-1 inhibitors, although these adverse events were reversible with drug discontinuation and/or corticosteroid therapy. One patient with a continuous complete response stopped the PD-1 inhibitor but maintained treatment with apatinib when the entire treatment lasted for 1 year. One patient discontinued anlotinib because of grade 3 throat pain. During the therapy, no patient underwent dose reduction of the anti–PD-1 inhibitors or anti-VEGF agents.

### Safety

Of the 43 patients for safety analysis, the median duration of exposure to combination therapy was 9.5 months (range, 0.5-24.8 months). Thirty-seven patients (86.0%) reported TRAEs of any grade. The most frequently reported events were fatigue, decreased appetite, and hypertension. The majority of the TRAEs were low grade. Grade 3 TRAEs occurred in 8 patients (18.6%). One patient developed grade 3 oropharyngeal pain, which was considered to be related to the small molecular anti-VEGF agent anlotinib. This patient discontinued anti-VEGF treatment for 1 month, and the pain stopped; thereafter, the patient refused to continue anlotinib, so only the immune checkpoint inhibitor was continued. One patient with treatment-related grade 3 increases in blood creatinine concentrations discontinued the anti–PD-1 monoclonal antibody toripalimab for 3.5 months; the abnormal creatinine concentration was relieved to grade 1 with corticosteroid treatment. There was no grade 4 or 5 TRAEs. Four patients discontinued treatment for TRAEs. All adverse events are shown in **Table 2**.

**Table 2:** Treatment-Related Adverse Events in all patients (n = 43).

Adverse events	Any grade	Grade 3*
Any	37 (86.0)	8 (18.6)
Fatigue	25 (58.1)	1 (2.3)
Decreased appetite	21 (48.8)	0
Hypertension	16 (37.2)	1 (2.3)
Hypothyroidism	14 (32.6))	1 (2.3)
Nausea	13 (30.2)	0
Increased blood glucose concentration	7 (16.3)	0
Oropharyngeal pain	7 (16.3)	2 (4.7)
Increased aminotransferase concentration	6 (14.0)	0
Pruritus	6 (14.0)	0
Anemia	5 (11.6)	0
Increased blood creatinine concentration	4 (9.3)	1 (2.3)
Bleeding	3 (7.0)	0
Reactive capillary hyperplasia	3 (7.0)	1 (2.3)
Infusion-related reaction	3 (7.0)	0
Hand-foot syndrome	2 (4.7)	1 (2.3)

Data are n (%) of all 43 participants. The table lists maximum grade adverse events reported at grades 1–2 in at least 5% patients and grade 3 events.

There were no grade 4 or 5 TRAEs.

### Efficacy

**Figure 1:**
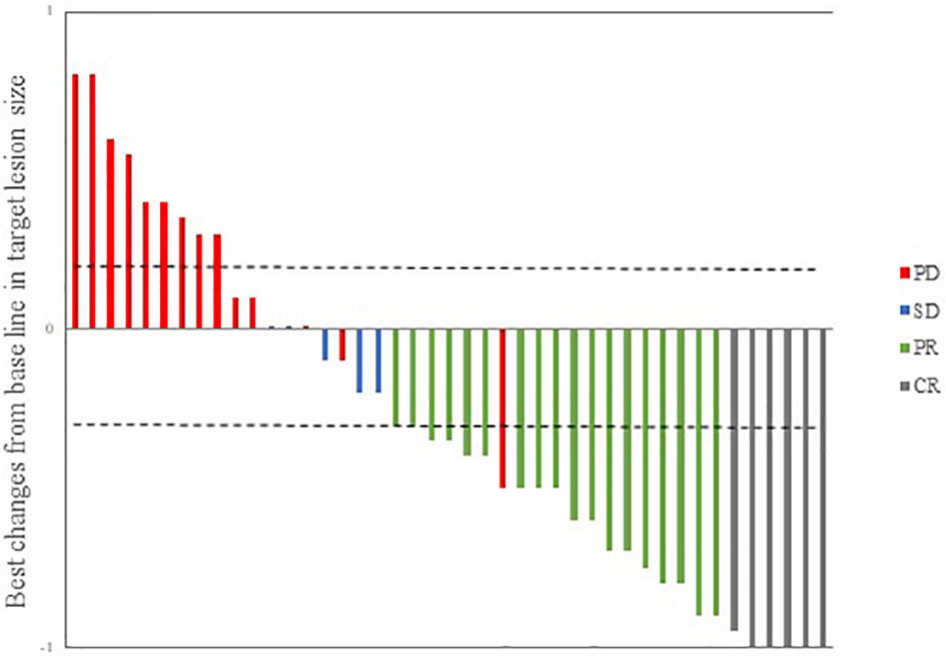
Waterfall plot illustrating maximum changes in target lesions size (n = 43). CR, complete response; PR, partial response; SD, stable disease; PD, progressive disease.

During follow-up, 24 patients (55.9%) had an ORR to treatment, and the DCR12 was 67.4%. The efficacy evaluation for all patients is shown in **Table 3**. According to the maximum percent change in target lesion size from the baseline, 25 patients experienced a response to the combination therapy; the corresponding waterfall plot is presented in **Figure 1**. The median time to response was 1.6 months (range, 1.1-2.8 months). Among these 25 responding patients, 24(96.0%) had an ongoing response at the last follow-up. Only 1 responding patient had subsequent disease progression. This patient had multiple hepatic metastasis but achieved an initial response after 1.7 months and then maintained treatment for 8.7 months; standard follow-up scans demonstrated a radiographic enlargement of hepatic disease, and the evaluation of curative effect was revised to progressive disease. At the data cutoff on March 31, 2021, the median DOR in responding patients was 10.5 months (range, 1.9-23.7 months).

**Table 3:** Clinical therapeutic evaluation for study patients.

All patients (n = 43)	
Response-evaluable patients	43 (100)
Overall response	
Complete response	6 (14.0)
Partial response	18 (41.9)
Stable disease	5 (11.6)
Progressive disease	14 (32.6)
ORR	24 (55.9)
DCR12	29 (67.4)

DCR 12, disease control rate 12; ORR, overall response rate.

**Figure 2:**
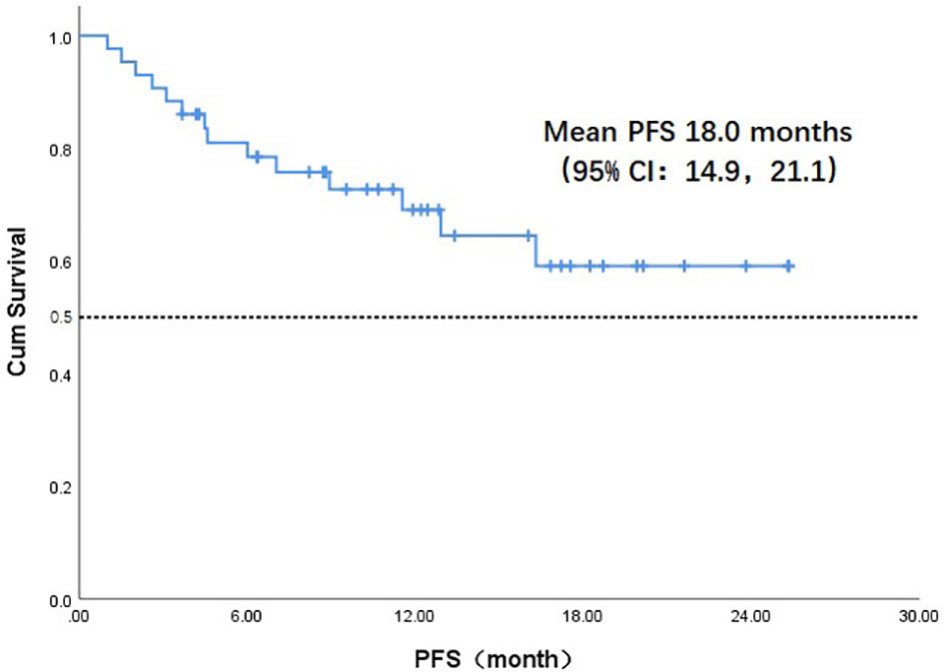
Kaplan-Meier survival curve of progression-free survival in 43 head and neck cancer patients. PFS, progression-free survival;CI, confidence interval.

**Figure 3:**
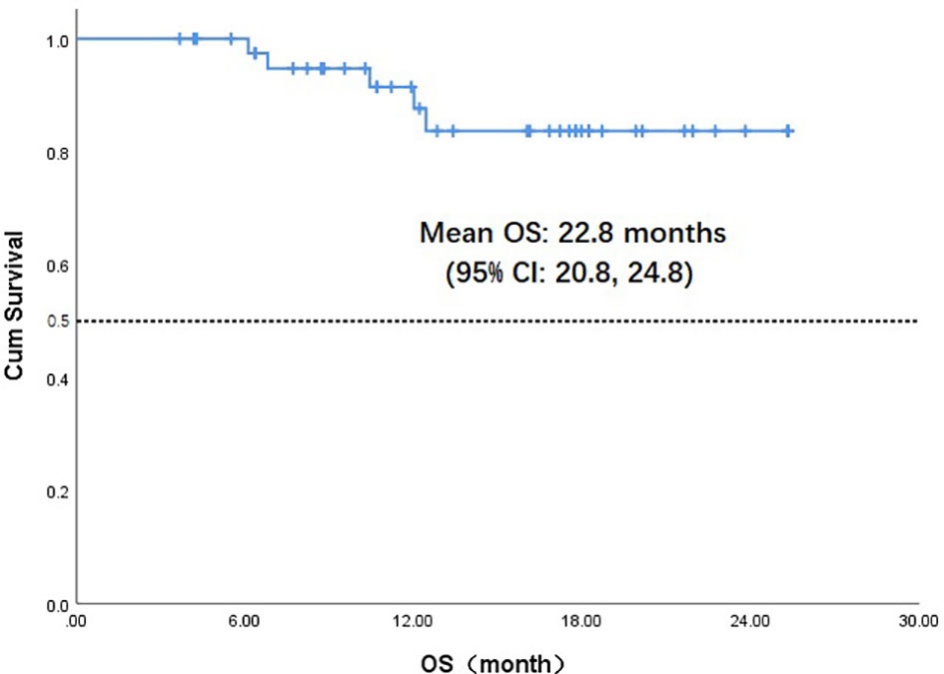
Kaplan-Meier survival curve of overall survival in 43 head and neck cancer patients. OS,overall survival;CI, confidence interval.

**Figure 4:**
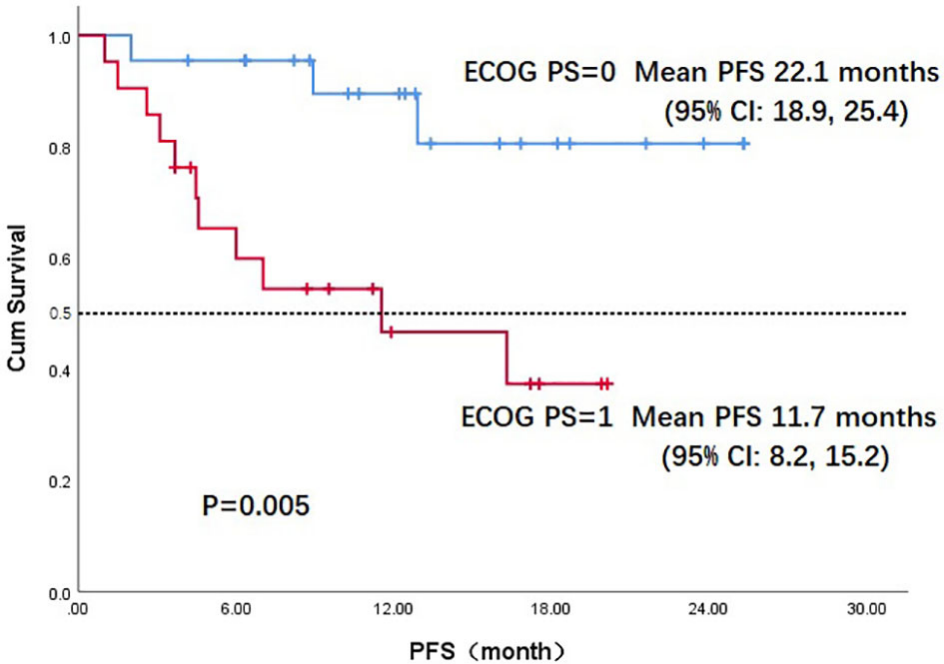
Kaplan-Meier survival curve of progression-free survival in ECOG PS 0 and PS 1 patients. PFS, progression-free survival;ECOG PS, Eastern Cooperative Oncology Group performance status; CI, confidence interval.

**Figure 5:**
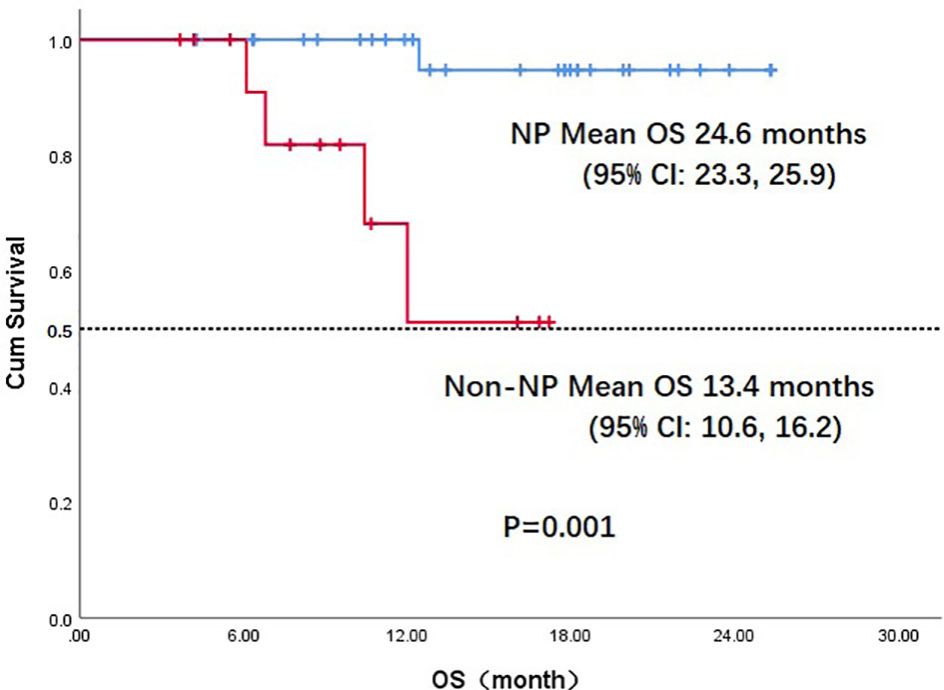
Kaplan-Meier survival curve of overall survival in NP and non-NP patients. OS, overall survival;NP, nasopharyngeal cancer; CI, confidence interval.

Throughout the treatment period, 10 patients developed disease progression, of which 3 tumors were at the site of the original focus, 4 were new lesions, and 3 were at both original and new sites. 4 patients developed disease progression after interrupting treatment for poor economic conditions. The median PFS was not reached, and the 1-year PFS was 69.1% (**Figure 2**). Five patients died from the disease during the observation period; the median OS was not reached, and 1-year OS was 87.7% (**Figure 3**). Univariate Cox analysis was performed to determine whether any clinical features were associated with PFS and OS. The ECOG PS score was related to PFS; patients with a PS score of 0 had a PFS advantage compared with patients with a PS score of 1 (p=0.005) (**Figure 4**). Patients with primary tumors located in the nasopharynx had better OS than those with tumors outside the nasopharynx, and the preponderance was significant (p=0.001) (**Figure 5**). Multivariate Cox analysis wasn’t performed because of the small sample size of the study. Details of the univariate analysis are shown in **Table 4**.

**Table 4:** Univariate analysis of factors associated with PFS and OS.

	PFS			OS		
	Mean ± SD (months)	χ^2^	p value	Mean ± SD (months)	χ^2^	p value
Age (years)						
≤60	19.6 ± 1.8	2.483	0.115	23.7 ± 1.0	2.149	0.143
>60	14.5 ± 2.9			20.6 ± 2.3		
Gender						
Male	17.9 ± 1.7	0.062	0.803	23.1 ± 1.0	1.078	0.299
Female	20.5 ± 4.2			18.6 ± 4.7		
ECOG PS						
0	22.2 ± 1.7	7.933	0.005	24.4 ± 0.9	2.917	0.088
1	11.7 ± 1.8			18.5 ± 1.0		
No. of prior systemic regimens						
1	NR			NR		
≥2	16.0 ± 1.8			22.2 ± 1.2		
Reasons for changed line						
Progression within 6 months	15.1 ± 2.6	0.139	0.669	23.1 ± 1.5	0.329	0.848
Progression after 6 months	17.3 ± 2.5			22.1 ± 1.6		
Intolerance	15.9 ± 2.6			19.9 ± 1.8		
Primary tumor						
Nasopharynx	19.5 ± 1.7	2.311	0.128	24.6 ± 0.7	10.422	0.001
Non-nasopharynx	10.9 ± 1.9			13.4 ± 1.4		
R/M at baseline						
Recurrence ± metastasis	12.2 ± 2.3	2.403	0.121	17.8 ± 1.5	0.434	0.510
Metastasis only	19.4 ± 1.7			23.2 ± 1.1		

ECOG PS, Eastern Cooperative Oncology Group performance status; NR, not reached (no event occurred); OS, overall survival; PFS, progression-free survival; R/M, recurrence and/or metastasis; SD, standard deviation.

## Discussion

Anti-angiogenic agents have the advantage of regulating the tumor immune microenvironment, so they are suitable to combine with immune checkpoint inhibitors. To date, VEGF is the most studied angiogenesis factor. Its immunosuppressive function manifests prominently as inhibition of immune effector cell differentiation and maturation (18). Many studies have observed that anti-VEGF agents promote the accumulation of CD8+ and CD4+ T lymphocytes in tumors, decrease PD-1 expression of tumor-infiltrating T lymphocytes and inhibit regulatory T cells as well as myeloid-derived suppressor cells and their immunosuppressive functions (23–25). However, angiogenic factors prevent immune cells from infiltrating into the tumor immune microenvironment through the tumor vessels. Overexpression of angiogenesis could promote expression of adhesion molecules and chemokines, and abnormal vascular structure could form a selective immune-cell barrier (26). Anti-angiogenic activity could promote the infiltration of different immune cells and upregulate the effectiveness of immunotherapy (27). Based on this mechanism, anti-VEGR agents in combination with anti–PD-1 monoclonal antibodies have been applied to lung cancer, hepatic cell cancer, and renal cell carcinoma; the combination was considered effective and tolerable, and no unexpected toxicities were observed.

In this study, we reviewed patients with R/M HNSCC who had experienced failure with or intolerance to frontline therapy to receive the combination strategy. In all, grade 3 TRAEs occurred in 18.6% of patients, and no grade 4 or 5 TRAEs occurred. Compared with monotherapy using PD-1 inhibitors or anti-VEGF agents, the combination strategy didn’t increase the incidence and severity of TRAEs, and no new TRAEs were observed. Moreover, the combination was associated with decreases in some specific adverse responses. The unique toxicity caused by camrelizumab was reactive capillary hyperplasia, which occurred at rates up to 74.1% in clinical studies of camrelizumab alone (28). In this study, 18 patients (41.7%) were treated with camrelizumab, but only 4 patients developed reactive capillary hyperplasia, accounting for 22% of patients taking camrelizumab. Zhou used camrelizumab and apatinib to treat patients with advanced *EGFR* and *ALK* wild-type non–small cell lung cancer; he found that the combination regimen significantly reduced reactive capillarity to 15.6%—a rate similar to that in our study (29). We speculate that anti-VEGF agents normalized the vascular malformation in skin and sodecreased the incidence and severity of reactive capillary hyperplasia.

Because of overlapping toxicities with combination therapy, it was difficult to determine whether the adverse events resulted from anti-VEGF agents or anti–PD-1 monoclonal antibodies. We can only classify them roughly according to our experience, which can help us deal with various adverse reactions. The 3 most common adverse events reported in this study, fatigue, decreased appetite, and hypertension, seemed related to antiangiogenic agents; however, some potentially immune-related adverse events, including hypothyroidism and increased blood glucose concentrations, were reported. The safety profile of the combination regimen was generally consistent with the known safety data of anti–PD-1 antibodies and antiangiogenic agents. No unique adverse events were reported in the HNSCC population. For 8 patients with grade 3 TRAEs, all were manageable by standard guidelines.

Twenty-four patients (55.9%) achieved overall responses in the study; this rate was less than the rates in the CAPTAIN-1st and JUPITER studies but more than the rate in Keynote048 (14, 30, 31). These 3 studies were designed to combine PD-1 inhibitors with systemic chemotherapy to treat first-line R/M HNSCC (including NPC). The ORR of our study was notable, because three quarters of patients had already received at least 2 lines of prior systemic therapy. At the data cutoff on March 31, 2021, the median DOR in responding patients was 10.5 months (range, 1.9-23.7 months); moreover, only 1 patient of the 25 experienced disease progression, which suggests that the DOR can be maintained for a long time after patients are in remission. Similar results have been observed with other combination strategies using PD-1 inhibitors and anti-angiogenic agents (e.g., in lung cancer, renal cancer). The success of combination strategies suggests that the immune checkpoint inhibitors combined with anti-angiogenesis agents might improve survival, not merely postpone treatment failure.

In our study, 22 patients (51.2%) had a PS score of 0. In univariate analysis, the PS score was associated with PFS; patients with a PS score of 0 had an advantage in PFS compared with those with a PS score of 1 (mean, 22.2 vs. 11.7 months, p=0.005). OS differed according to the location of primary tumor: the OS in patients with nasopharyngeal carcinoma was much better than in those with non-nasopharyngeal carcinoma (mean, 24.6 vs. 14.9months, p=0.027); the difference was attributed to the prognosis difference between the tumor types. The combination of PD-1 inhibitors and anti-angiogenic agents has increased the 1-year OS from a range of 36%-60% to 92% according to data from Checkmate-141 and BGB-A317-102, which used anti–PD-1 monotherapy in later treatment lines for R/M HNSCC and nasopharyngeal carcinoma (10, 32). These results suggest that the antitumor efficacy of a combination strategy is at least additive and possibly synergistic. In our study, the antitumor activity of the combination regimen was superior to that expected from anti–PD-1 monotherapy.

However, this study had some limitations. First, this study was retrospective, so its intrinsic selection bias may be responsible for the observed differences in outcome. Randomized comparisons between PD-1 inhibitors alone and PD-1 inhibitors in combination are warranted to avoid imbalances and selection biases. Second, the relatively small sample size of the patient cohort might weaken the effectiveness of the statistical analysis. Last, 6 PD-1 inhibitors and 3 VEGF inhibitors were utilized. As different agents had different molecular targets and toxicity profiles, which would make the analysis of efficacy and safety more difficult.

## Conclusion

This case-series study shows that the combination of anti–PD-1 monoclonal antibodies and anti-VEGF agents is tolerable in patients with R/M HNSCC and that the treatment exhibits antitumor potential despite the heavily pretreated status of many enrolled patients. The path from laboratory to clinic remains long; in-depth research should be investigated according to different primary tumors, and the mechanism of the synergistic effects of anti–PD-1 and anti-VEGF agents must be explored.

## Data Availability Statement

The raw data supporting the conclusions of this article will be made available by the authors, without undue reservation.

## Ethics Statement

The studies involving human participants were reviewed and approved by Ethics Committee of Zhejiang Cancer Hospital. The ethics committee waived the requirement of written informed consent for participation.

## Author Contributions

Conception and design: YH and XC. Collection and assembly of data: YH, QJ, and TJ. Data analysis and interpretation: YH and RD. Manuscript writing and final approval of manuscript: all authors. All authors contributed to the article and approved the submitted version.

## Funding

This work was supported by the Zhejiang Natural Science Foundation (LY17H160042), and the Medical Science and Technology Project of Zhejiang province (2016ZB021).

## Conflict of Interest

The authors declare that the research was conducted in the absence of any commercial or financial relationships that could be construed as a potential conflict of interest.

## Publisher’s Note

All claims expressed in this article are solely those of the authors and do not necessarily represent those of their affiliated organizations, or those of the publisher, the editors and the reviewers. Any product that may be evaluated in this article, or claim that may be made by its manufacturer, is not guaranteed or endorsed by the publisher.
